# Bulk transcriptomics of peripheral blood mononuclear cells delineates systems-level immune dysregulation in pediatric dengue infection

**DOI:** 10.1038/s41540-026-00796-y

**Published:** 2026-08-14

**Authors:** Abdul R. Anshad, Muthuvel Atchaya, Amudhan Murugesan, Suvaiyarasan Suvaithenamudhan, Shanmugam Saravanan, Sivadoss Raju, Cecilia Svanberg, Marie Larsson, Esaki M. Shankar

**Affiliations:** 1 Infection and Inflammation, Department of Biotechnology, Central University of Tamil Nadu, Thiruvarur, India; 2 Department of Microbiology, The Government Theni Medical College and Hospital, Theni, India; 3 School of Biosciences and Bioengineering, D. Y. Patil International University, Pune, Maharashtra India; 4 Dental Research Cell , Dr. D. Y. Patil Dental College and Hospital, Dr. D. Y. Patil Vidyapeeth, Pune, Maharashtra India; 5 State Public Health Laboratory, Directorate of Public Health and Preventive Medicine, DMS Campus, Chennai, India; 6 Molecular Medicine and Virology, Department of Biomedical and Clinical Sciences, Linköping University, Linköping, Sweden

**Keywords:** Computational biology and bioinformatics, Diseases, Genetics, Immunology

## Abstract

Children are disproportionately at a greater risk of developing severe dengue disease, yet the molecular mechanisms driving the heightened susceptibility remains poorly understood. To elucidate the immune determinants of pediatric dengue pathogenesis, we profiled the transcriptomic landscape by performing RNA sequencing (RNA-Seq) on peripheral blood mononuclear cells (PBMCs) from laboratory-confirmed cases of primary and secondary pediatric dengue, as well as pediatric healthy controls. Differential gene expression (DEG) and pathway analyses were performed to delineate the transcriptional alterations. Pediatric dengue was marked by extensive transcriptional changes with altered expression of genes associated with the complement cascade macrophage-associated genes as well as immune checkpoint molecules. Notably, secondary infection had significant alterations in immune checkpoint molecules and genes associated with macrophage activation, suggesting the onset of immune exhaustion during reinfection. Differences between the primary and secondary dengue cohorts were modest relative to the transcriptional differences observed between either of the groups and healthy controls, with immune checkpoint genes showing the most consistent divergence between the two dengue cohorts. While our initial findings provide early insights into the transcriptional patterns potentially associated with disease outcomes, the dysregulated transcriptomic signatures reported here are preliminary requiring systemic functional validation to determine their biologic role in the immunopathogenesis of pediatric dengue infection.

## Introduction

Dengue represents a serious global public health challenge. The rapidly spreading mosquito-borne viral disease appears to have caused ~390 million infections annually across over 100 countries, particularly in the tropical and subtropical regions^1,2^. Seasonal outbreaks coincide with the pre-monsoon season and are driven by female Aedes mosquitoes transmitting four antigenically distinct dengue virus (DENV) serotypes, DENV1-4^1,3^. Each serotype comprises multiple genotypes or clades based on their epidemic potentials and geographical distribution^4^. While primary dengue often remains asymptomatic or subclinical, secondary infection with the same serotype provides lasting immunity. However, secondary infection with heterologous serotypes of DENV can result in dengue hemorrhagic fever (DHF) and dengue shock syndrome (DSS), which may have far-reaching consequences. This mechanism involves antibody-dependent enhancement (ADE) often driven by non-neutralising antibodies resulting in enhanced viral replication and pathogenesis. In addition, immune imprinting (also called original antigenic sin (OAS)) can alter the immune response by selectively activating cross-reactive memory cells during secondary infection^5,6^. Hence, ADE and immune imprinting appear to shape disease outcome in secondary dengue^6–9^. Importantly, the sequence of infections with DENV serotype(s) significantly influence dengue disease outcomes, with certain serotype combinations conferring higher risk of severe disease^10,11^.

DENV genome represents a linear, non-segmented, positive-sense ssRNA (ssRNA + ). DENV open reading frame (ORF) has three structural proteins viz., the capsid, membrane/pre-membrane, and envelope as well as seven non-structural (NS) proteins (i.e., NS1, NS2A, NS2B, NS3, NS4A, NS4B, and NS5)^12,13^. Notwithstanding the virus continues to evolve, adaptive mutations remain masked by wild sequences, which become detectable upon assuming dominance^14^. Evidence suggests that the risk of severe dengue is contingent on both the infecting serotype as well as the sequence of infection with heterologous virus serotypes^10,15^.

Severe dengue remains a leading cause of childhood mortality^16^, and therefore merits investigations to unravel the complexities underlying host-pathogen interactions. According to the 2009 WHO classification, dengue is categorized into severe and non-severe infections. The non-severe form is further differentiated based on disease signs and symptoms into dengue with warning signs (DWS + ) and dengue without warning signs (DWS-)^17,18^. During DENV infection, transcriptomic profiles in immune cells hint at the expression of diverse host gene signatures that predict disease severity and disease progression, also aiding in early diagnosis and management. Moreover, RNA sequencing (RNA-Seq) aids in the characterisation of transcriptomic changes, extending improved insights into immune activation, immune dysregulations, complement activation, acute-phase reactants and cellular biochemical pathways that distinguish between disease manifestations^19,20^. Literature concerning transcriptomic profiling between primary and secondary pediatric dengue infection appears to be scarce^21–24^.

Here, we conducted a pilot study to explore the differential gene expressions (DEG) of key immune mediators in bulk PBMCs of pediatric subjects with primary and secondary dengue, as well as pediatric healthy controls. Our findings provide preliminary insights into the immunopathogenesis of pediatric dengue disease.

## Results

### Study outline and demographic characteristics

Pediatric DENV-infected patients and healthy control were enrolled (*N* = 9), six dengue patients with three primary dengue, three secondary dengue, and three healthy volunteers aged between 3 and 12 years of age. Clinico-demographical data were collected. RNA was purified from PBMCs and used for RNA sequencing followed by data analysis. Five patients were diagnosed with dengue with warning signs (DWS + ), and one individual was diagnosed with dengue without warning signs (DWS−). All six dengue patients were febrile and myalgic at the time of sampling, whereas headache was reported in five patients. Rash and bleeding manifestations were not evident in any of the patients studied, and arthralgia was reported in two pediatric patients (Suppl. Table 1).

### Patients with secondary pediatric dengue presented comparable biochemical parameters with that of pediatric subjects with primary dengue infection

**Fig. 1:**
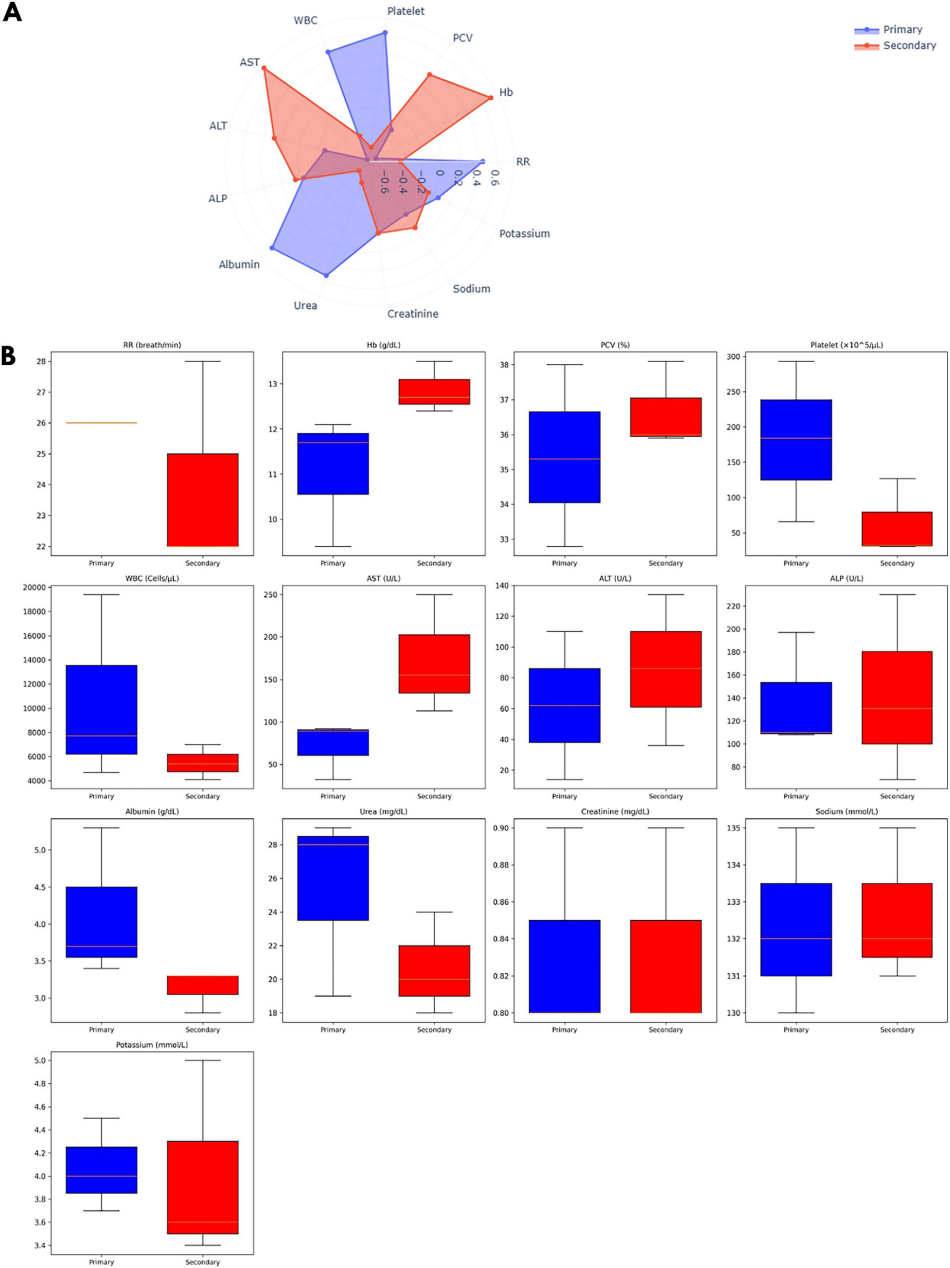
Physiological, biochemical and laboratory parameters in primary and secondary pediatric dengue infections. Comparative distribution of biochemical and hematological parameters in primary and secondary pediatric dengue infections. **A** Overall depiction of the relative variation of the blood parameters between primary and secondary pediatric dengue. **B** Clinical analytes among pediatric patients with primary and secondary dengue.

The clinicodemographic and laboratory parameters did not show any significant difference between the primary and secondary groups. The respiratory rate, renal function parameters, and haematological indices, and other parameters did not differ between the primary and secondary pediatric dengue cohorts (Suppl. Table 1). Of the liver parameters, AST showed a significant elevation in the secondary dengue cohort (*p* = 0.086), while there were no significant differences in ALP and ALT levels between the primary and secondary DENV cohorts. Serum albumin levels, renal function as well as electrolytes also did not show significant alteration between secondary dengue and primary dengue (*p* > 0.05). In addition, platelet count was reduced, albeit not significantly, in the secondary dengue vs primary dengue cohort (Fig. 1).

### Primary component analysis (PCA) revealed that the transcriptomic landscape of DENV-infected pediatric subjects was distinct from their uninfected counterparts

We conducted a PCA to explore the global transcriptional variations among healthy controls, and the primary and secondary DENV cohorts (Suppl. Fig. 3). The first two principal components explained a cumulative 62% total variance (PC1 = 37% and PC2 = 26%), effectively separating the DENV-infected from the controls into distinct clusters. The three HCs clustered on the negative side of the PC1 axis indicating a homogeneous transcriptional profile in the absence of DENV infection. On the contrary, both the DENV-infected groups clustered on the positive side of PC1, clearly distinguishing from the HCs. We did not observe any distinct cluster in transcriptional responses between the primary and secondary dengue cases. Together, the PCA demonstrates that primary and secondary dengue infections are transcriptionally distinct from HCs (Suppl. Fig. 3).

### Pediatric dengue infection showed marked downregulation of mRNA associated with several proinflammatory mediators but not interferon-driven genes

To identify the transcriptional signature associated with immune responses in pediatric dengue infection, we compared the transcriptome data across all the three study cohorts. The DEG analysis (with adjusted *p*-value [FDR] < 0.05 and |log_2_FC | ≥2) revealed 1539 and 1937 DEGs in primary and secondary pediatric dengue, with an upregulation of 746 and 1012 genes, respectively (Suppl. Table 2). There was a substantial overlap of DEGs between the primary and secondary dengue infections (Note: the overlap count has been corrected based on re-examination of the Venn diagram data) (Suppl. Fig. 4). Across all the cohorts, the DEG population was dominated by the protein-coding transcripts, followed by long non-coding RNAs. We also observed the presence of pseudogenes and small RNAs in secondary pediatric dengue infection (Suppl. Fig. 5).

Of the top 25 protein-coding genes in primary and secondary DENV infection (Suppl. Fig. 6), we observed an upregulation in *CH25H, ARG1*, and *ADAMTS2* and a concurrent downregulation of neutrophil *DEFA1/3/* in primary dengue infection. In secondary dengue, the expression of *ACOD1* (*IRG1*) and neutrophil-associated genes were reduced alongside the upregulation of *ADAMTS2*. As compared with the primary, secondary dengue infection showed evidence for the downregulation of anti-inflammatory and endothelial-protective genes such as *ACOD1* and *TFPI2*, alongside the upregulation of pro-inflammatory and remodelling genes including *IL21, IGFBP2*, and *COL16A1* (Suppl. Fig. 7).

**Fig. 2:**
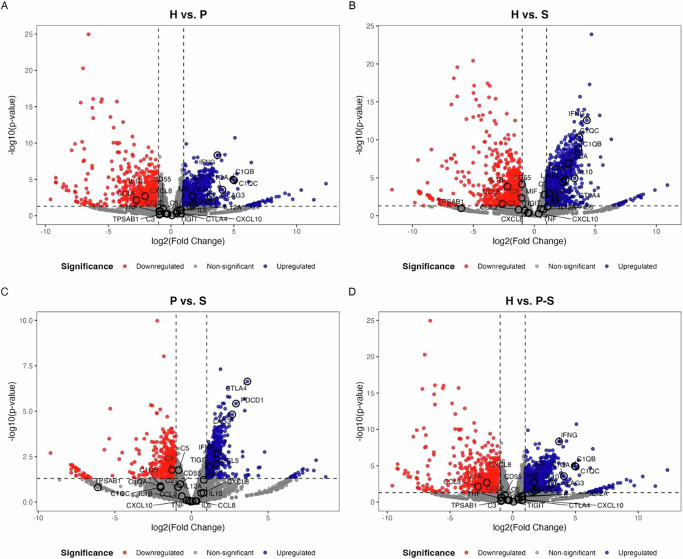
Distinct immune transcriptional signatures in primary, and secondary pediatric dengue compared to healthy controls. Differential expression analysis of genes in (**A**) Primary dengue vs Healthy (H vs P). **B** Secondary dengue vs Healthy (H vs. S). **C** Primary dengue vs Secondary dengue (P vs. S). **D** Healthy vs dengue: Each point represents a gene, with the log₂ fold change (log₂FC) on the x-axis and the –log₁₀ (*p*-values) adjusted *p*-value (FDR) on the y-axis. Genes significantly upregulated (log₂FC > 1, –log₁₀ (*p*-values) < 0.05) are shown in red, while significantly downregulated genes (log₂FC < –1, –log₁₀ (*p*-values < 0.05) are shown in green. Non-significant genes are depicted in grey. Vertical dashed lines indicate the ±1 log₂FC thresholds, and the horizontal dashed line marks the –log₁₀ *p*-value significance cut-off (0.05). Labelled genes represent some of the genes of interest.

Between the primary dengue and HCs, proinflammatory mediators *IL1B, TNF*, and *CCL2* were downregulated, while *IFNG*, and *CXCL10* as well as complement components *C1Q A–C* were upregulated akin to immune checkpoints *PDCD1* and *LAG3* (Fig. 2A). On the other hand, between secondary dengue and HCs, a similar pattern was observed with additional induction of *IL10, CCL5*, and *C2* (Fig. 2B). Between secondary and primary DENV infections, secondary pediatric dengue showed stronger upregulation of *PDCD1, LAG3*, and *IFNG*, alongside greater suppression of *IL1B* and *TNF* (Fig. 2C). The combined dengue vs HCs also reflected a similar DEG as observed in the primary as well as secondary dengue infections (Fig. 2D).

### Primary pediatric dengue unveiled the potential role of gene signatures associated with complement activation

**Fig. 3:**
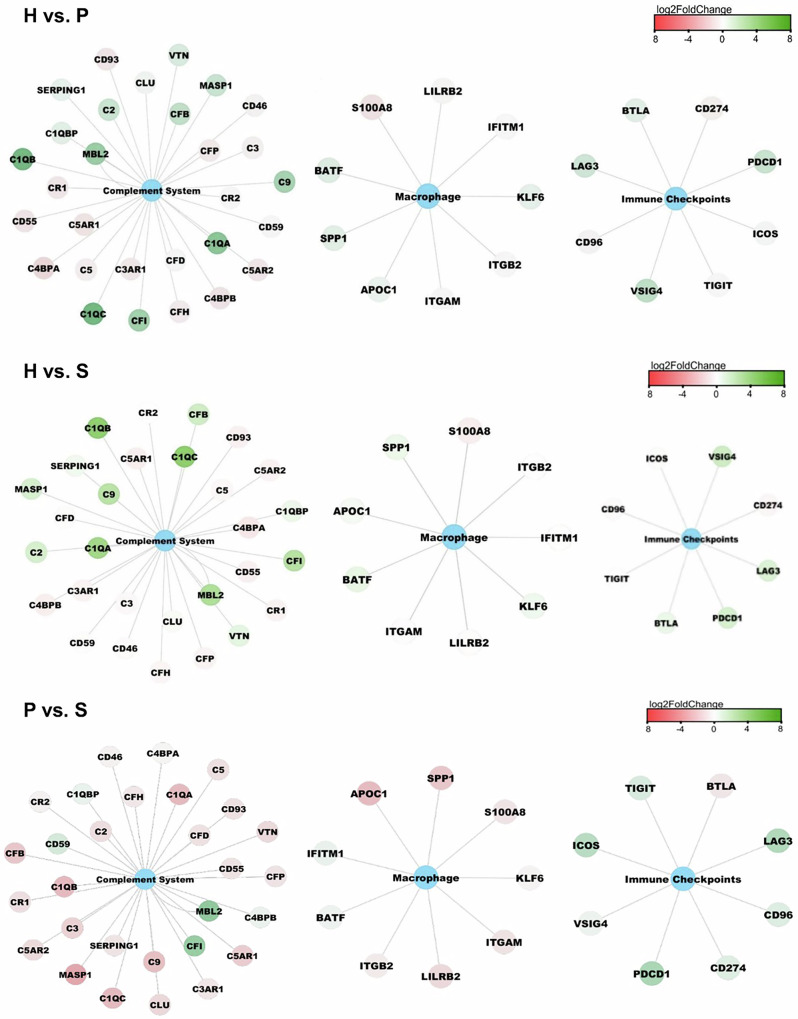
Functional network of differentially expressed genes. **A** Primary pediatric dengue vs healthy controls (H vs. P). **B** Secondary pediatric dengue vs. healthy controls (H vs. S). **C** Primary pediatric dengue vs Secondary pediatric dengue (P vs. S). Nodes represent genes, and edges represent functional associations. Blue nodes denote hub genes/pathways, while green and red nodes indicate upregulated and downregulated genes, respectively. The network highlights dysregulation of complement, macrophage associated genes and immune checkpoints in pediatric dengue patients.

Our DEG analysis revealed distinct regulation of multiple components of the complement system. Multiple classical and lectin pathway genes were upregulated in the primary dengue cohort, including *MBL2, C1QA, C1QB, C1QC, C2*, and *C9* (Fig. 3). In contrast, several complement regulatory factors were downregulated in the primary dengue cohort, including *CD93, properdin (CFP), CR1, SERPING, C4BPA, C4BPB*, and *CFD* (Fig. 3).

### Relative abundance of gene signatures associated immune checkpoints and acute phase response was imminent in primary pediatric DENV infection

We found that the primary dengue cohort exhibited significant enrichment of genes associated with immune checkpoints, including *PDCD1, LAG3, ICOS*, and *VSIG4*, while *LAG3, CD96*, and *BLTA* that were downregulated. In addition, primary dengue also had alterations in acute-phase response genes. *ITGAM, ITGB2, SPP1, APOC1, LILRB2,* and *IFITM1* were upregulated while *BATF and S100A8* subsided in primary dengue (Fig. 3).

### Biosignatures associated with complement activation and macrophage-associated genes were evident in secondary pediatric dengue disease

The DEG analysis of the complement pathway in the secondary dengue cohort followed a similar pattern similar to primary dengue whereas the complement regulators were markedly downregulated. Similarly, in secondary dengue cohort, macrophage associated marker *ITGB2, SPP1, LILBR2, AFIT1M, and KLF6* were upregulated while *S100A8* and BATF were downregulated, which aligns with what we observed in the primary dengue cohort (Fig. 3). Moreover, immune checkpoints associated genes VSIG4, PDCD1, ICOS, and CD274 were upregulated whereas *LAG3, BTLA, TIGIT*, and *CD96* exhibited downregulation.

### Comparative RNA-Seq atlas revealed the role of complement activation and regulatory cytokines in secondary as compared to primary pediatric dengue

Next, we compared the secondary with the primary dengue cohort, which secondary exhibited DEG profiles wherein the complement cascade exhibited a substantially higher dysregulation. We note that after correction for multiple testing (FDR < 0.05, |log₂FC | ≥ 2), none of the complement-pathway genes examined showed statistical significance in the direct primary-versus-secondary comparison (Supplementary Table 3); the differences described are based on nominal expression trends rather than FDR-significant differential expression, and should be interpreted accordingly. The module revealed broad down-regulation of classical (*C1QA, C1QC, C2, C4B*), alternative (*CFB, CFD, CFH*), lectin (*MASP1*) and terminal (*C5, C9*) components along with regulators (*C4BP, CFI, CR1*/*CD35*) in secondary dengue. In contrast, recognition molecules such as *CFI* and *MBL2* were upregulated. In parallel, the macrophage-associated module shows upregulation of *ITGB2, SPP1*, and *KLF6* while immune checkpoints-mediated immune regulation displayed enrichment of inhibitory molecules (*VSIG4, TIGIT, CD96*, and *LAG3*) (Fig. 3).

### GO enrichment analysis showed evidence for enrichment of immune and regulatory pathways across the primary and secondary dengue infections

**Fig. 4:**
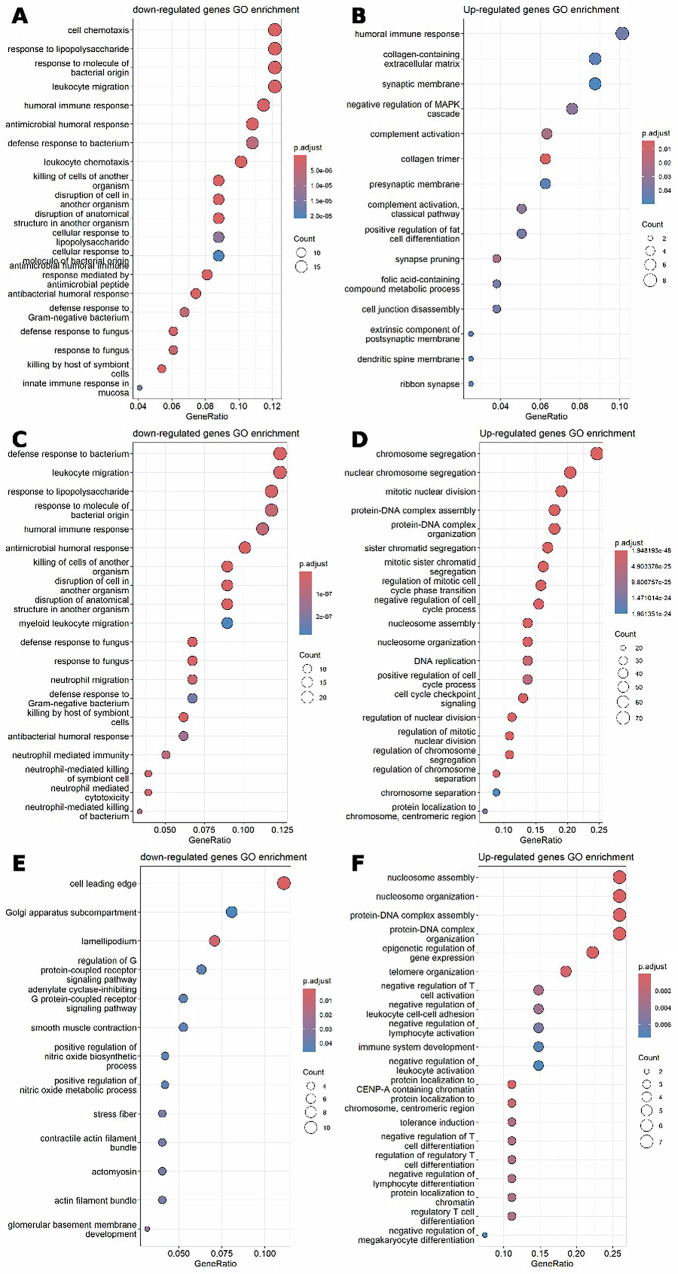
Gene ontology (GO) enrichment analysis of differentially expressed genes in dengue infection. The bubble plot represents enriched GO biological process for down and upregulated genes across primary and secondary dengue. Panels (**A**, **B**) show enrichment for primary dengue vs healthy controls, panels **C** and **D** represents secondary dengue vs healthy controls, and panels (**E**, **F**) corresponds to primary vs secondary dengue. Panels (**A**, **C**, **E**, **B**, **D**, **F**) represents the down regulated and upregulated genes, respectively. The x axis indicates the gene ratio, while the color represents the adjusted *p*-value.

In the primary dengue cohort, we observed a significant downregulation in the genes associated with immune and inflammatory processes such as leukocyte chemotaxis, humoral responses, and many other physiological pathways (Fig. 4A). Conversely, the upregulated genes in the primary dengue vs healthy were mostly enriched in the pathways associated with the complement activation, matrix organization, synaptic signalling (Fig. 4B).

Of the genes upregulated in the secondary dengue cohort were those associated with cell cycle related processes such as chromosome segregation, mitotic division and DNA replication while genes involving in leukocyte migration, and innate immune activation were downregulated (Fig. 4C, D). Comparative analysis between primary and secondary dengue further highlighted dynamic immune regulation, with genes involved in the cytoskeletal organization and NO biosynthesis (Fig. 4E). At the same time, upregulated genes were enriched in nucleosome assembly, epigenetic regulation, and regulation of T-cell activation and differentiation (Fig. 4F).

### Pathway analysis demonstrated the potential role of mediators linked to innate immune activation during secondary pediatric dengue infection

**Fig. 5:**
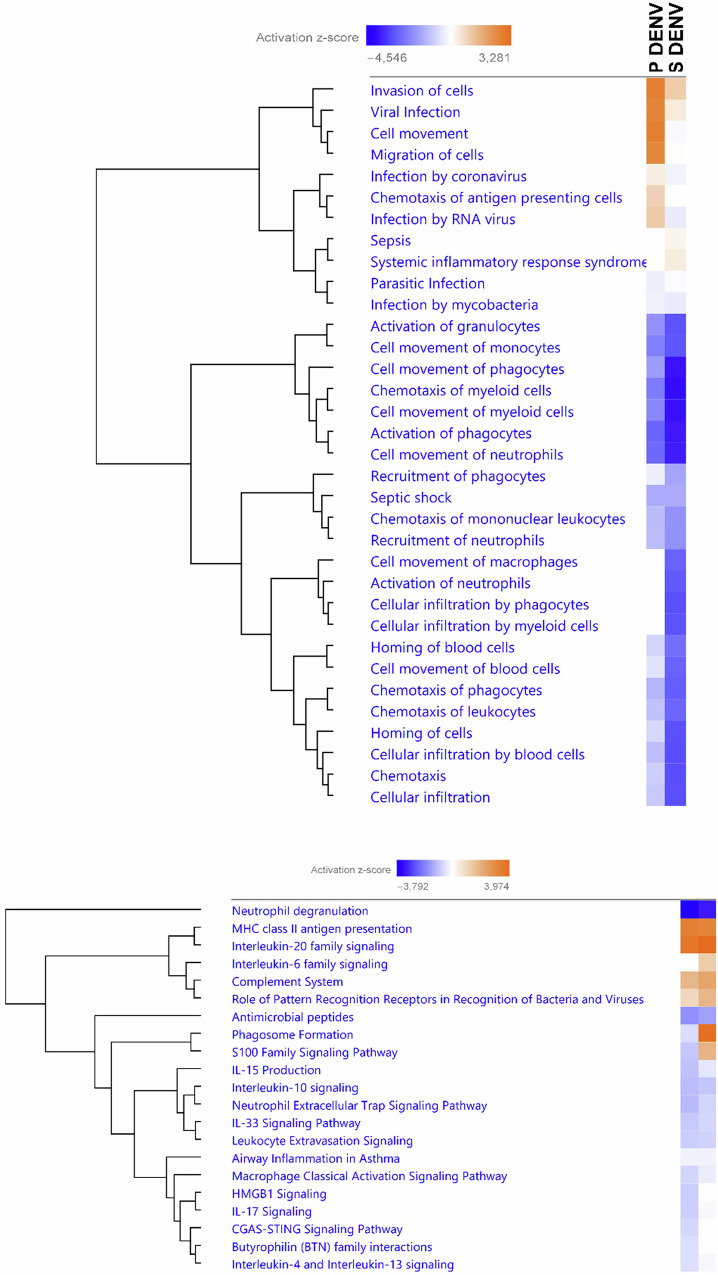
Canonical pathways activated in primary and secondary pediatric DENV infection. The Z-score reflects the direction and magnitude of pathway regulation, with orange indicating predicted activation and blue indicating predicted inhibition. Pathways are hierarchically clustered according to similarity in their activation patterns across experimental groups, highlighting coordinated regulation of key immune processes.

Pathway analysis (IPA) predicted activation of immune processes including neutrophil degranulation, MHC class II antigen presentation, and multiple interleukins signalling pathways, as well complement cascade activation. These pathways are crucial for anti-viral sensing and inflammation. In the secondary dengue cohort, we observed an increased predicted activation of mediators associated with phagocytosis, S100 family signalling, and classical macrophage activation, indicating a more robust or dysregulated response compared to primary infection. Disease or biofunction-enriched cellular movement, chemotaxis, and cellular infiltration are the hallmarks of inflammation and immune cell recruitment. The most definitive contrast between DEGs in primary and secondary dengue included granulocyte activation, phagocyte recruitment, and chemotaxis, which were suggestive of an increased risk for exacerbation of disease symptoms in secondary dengue (Fig. 5). We emphasise that IPA pathway activation z-scores and GO enrichment terms are computational predictions derived from the directionality and magnitude of differentially expressed transcripts. They indicate the statistically over-represented pathways and the direction in which they are predicted to be regulated, rather than constituting direct biochemical or functional evidence of activity. These predictions should accordingly be regarded as hypothesis-generating and require independent validation (e.g., by qRT-PCR, ELISA-based pathway readouts, or functional assays) before being interpreted as evidence of genuine pathway activation.

## Discussion

DENV infection displays a dynamic epidemiological pattern in India, characterized by its seasonal occurrence, increasing incidence of secondary infections, and diverse clinical manifestations across the four viral serotypes^25,26^. A deeper understanding of host–pathogen interactions is needed to identify key determinants of disease pathogenesis. Furthermore, unravelling the viral genome and transcriptome will aid in the development of novel drug targets and preventive modalities against DENV. In this study, we present a comprehensive transcriptomic profiling of pediatric DENV infection, delineating the interplay between complement activation, acute-phase and IFN responses, metabolic remodelling, and cellular signaling networks. Across all the cohorts, DEG population was dominated by the protein coding transcripts, followed by long non-coding RNAs, suggesting broad transcriptional alterations involving both coding and regulatory non-coding RNAs play role in dengue pathogenesis. Further, the presence of pseudogenes and small RNAs implies the genes highlighting additional transcriptional complexity in secondary DENV infection. Our data highlights the intricate immune transcriptional landscape involved in pediatric dengue pathogenesis. Our PCA analysis and hierarchical clustering demonstrated potential transcriptional segregation between dengue-infected and healthy pediatric subjects, hinting extensive immune activation following infection. However, the overlapping genes that correspond to primary and secondary dengue appear to suggest a shared antiviral response, with the secondary dengue marked by amplified inflammatory modules. The substantial overlap observed between primary and secondary dengue cohorts suggests shared host responses, although a limited subset of unique transcripts reflect the biologically relevant differences associated with prior history of infection. Furthermore, the observed differential expression of lncRNAs, pseudogenes, and other non-coding RNA should be considered descriptive transcriptomic observations, as their functional significance cannot be established solely from their expression.

The distinctive transcriptomic landscape observed in the dengue-infected pediatric cohort suggests a coordinated immune response characterised by proinflammatory mediators, type I IFN-driven responses, and immune checkpoint–regulated pathways. The biological relevance of several of the genes to dengue pathogenesis warrants further discussion. *CH25H* encodes an IFN-stimulated cholesterol-25-hydroxylase that restricts entry of several enveloped viruses through production of 25-hydroxycholesterol^27^, consistent with an IFN-driven antiviral program in primary dengue. *ARG1* encodes arginase-1, a marker of alternatively activated myeloid cells linked to suppression of antiviral T-cell responses in other viral infections, including SARS-CoV-2 and certain arthritogenic alphaviruses^28^. To our knowledge, its role in dengue has not been previously described and represents a novel candidate warranting dedicated investigation. *ADAMTS2* is a procollagen N-proteinase involved in extracellular matrix remodelling; although ADAMTS2 itself has not previously been reported in dengue, its upregulation aligns with broader reports of extracellular matrix and cell-junction gene dysregulation during DENV infection^29^. The downregulation of neutrophil-derived α-defensins *DEFA1/DEFA3* mirrors findings in a pediatric dengue cohort in which neutrophil-derived antimicrobial peptide expression was linked to clinical severity^30^. *ACOD1* (*IRG1*) generates the immunometabolite itaconate and restricts flavivirus (Zika virus) replication in neurons by inhibiting succinate dehydrogenase^31^; its downregulation in secondary dengue likely indicates an attenuated cell-intrinsic metabolic antiviral response, although this remains to be examined in DENV infection, specifically. *TFPI2* is a Kunitz-type protease inhibitor with established roles in extracellular-matrix stabilisation and anti-angiogenic activity^32^. *IL21* has previously been reported among the transcriptional signatures of DENV-specific CD4 T-cell subsets during acute infection^33^, supporting a role in adaptive immune regulation during secondary dengue. By contrast, *IGFBP2* and *COL16A1* have not, to our knowledge, been previously implicated in dengue infection; we report these as novel candidate genes whose functional relevance to pediatric dengue pathogenesis remains to be established.

In particular, dysregulation in the complement system, a key arm of innate immune responses, was apparent across the cohorts. In primary dengue, several classical and lectin pathway mediators were upregulated, implying an initial complement activation^34–36^. We use the term “activation” here to describe the direction of differential gene expression rather than direct biochemical evidence of complement cascade activity, which transcriptomic data alone can seldom establish. Further analysis of the primary vs secondary dengue cohort data revealed a widespread downregulation of *C1QA, C1QC, C2*, and *C4b* accompanied by upregulation of *CFI, C4BP, MBL2,* and *CFP*, which suggests a transition from activation to regulation, likely reflecting a standby compensatory mechanism to limit complement-mediated inflammation and tissue injury during secondary dengue infection^37^. This is supported by the upregulation of CFI and C4BP, which align with the known phenomenon of immune exhaustion or feedback suppression during severe dengue^38^. Complement activation in dengue is reportedly a double-edged sword, as even though it aids in viral neutralization, inasmuch as excessive or unbalanced activation can contribute to vascular permeability, plasma leakage, which are pathognomonic hallmarks of severe dengue^36,39,40^. The upregulation of macrophage-associated markers (*ITGb2, SPP1*, and *KLF6*) across both primary as well as secondary dengue affirms robust macrophage activation and tissue remodelling. The downregulation of *S100A8* and *BATF* appears to represent feedback restrain exaggerated inflammation. These transcriptomic observations are suggestive of a role for the monocyte-macrophage axis, pending functional confirmation^41^.

Immune checkpoint, including *PDCD1, LAG3, TIGIT, CD96*, and *VSIG4*, were upregulated in both primary and secondary pediatric dengue, with markedly higher expressions in the secondary cohort. This enhanced checkpoint activity aligns with the immune exhaustion observed in NK cells, MAIT cells, and T cells, which restrict antiviral effector function^42,43^. These features highlight their role disease progression^44,45^.

Gene ontology and pathway enrichment analyses further demonstrated that primary dengue is dominated by inflammatory genes while secondary dengue mainly feature an enrichment of cell cycle, nucleosome assembly, and epigenetic regulatory pathways suggesting enhanced cellular activation and proliferative remodelling in severe infection^46,47^. Notably, our findings also highlight the involvement of IL17A in driving coordinated inflammatory and vascular responses in secondary DENV infection. The prominent links between cytokines (*IL1A, IL1B*, and *TNF*) and downstream cellular processes reinforcing excessive immune activation leads to heightened cellular movement and possible tissue damage via ROS^48,49^. We propose mechanisms linking RIG-1, TLR, and IFN signalling pathways in the regulation of dengue-associated immune mediators. Several key transcriptional regulators exhibited differential expression, where the RIG-1, TLR, IFN signalling pathways reveal a coordinated regulation of innate immune response leading to inflammatory and stress-associated outcomes. Upon entry of DENV into the host cell, the RIG-1 receptors will sense DENV-derived ssRNA that induces the expression of downstream intermediates. Activation of the signalling cascades culminates in the production of proinflammatory cytokines, chemokines and type 1 IFNs that will orchestrate recruitment of immune cells and effectors. This cytokine and chemokine-derived responses stimulate the synthesis of acute phase reactants, which in turn activates the complement cascade reinforce viral clearance as well as amplification of inflammatory cascades. In parallel, cytokines and chemokines will foster oxidative and ER stress reflecting cellular adaptation to heighten metabolic and proteotoxic load. Further, metabolic remodelling genes signify a shift toward immunometabolism reprogramming. Moreover, immune checkpoint expression culminates in skewing a modulation towards T-cell exhaustion and immune regulation. Overall, the differential regulation of RIG-1/TLR/IFN signalling component leads to multifaceted responses characterized by cytokine and chemokine secretion, acute phase responses, and metabolomic stress reprograms together shaping tightly controlled inflammatory environment during dengue infection. The genes highlighted in red denotes downregulated genes whereas that in blue indicates upregulated genes. This model is derived from transcriptomic data and requires experimental validation through qRT-PCR, flow cytometry, and functional assays.

Being a pilot study, our investigation has certain limitations that warrant due consideration. Firstly, dengue viral serotyping was not performed, which limits our capacity to distinguish serotype-specific transcriptomic effects from those attributable to infection history (primary vs secondary). Given that the sequence of infection with DENV serotypes significantly influencing disease severity and immune responses^10^, future studies should incorporate serotyping to holistically contextualize the transcriptomic findings. Secondly, the small sample size (three per group) renders our study susceptible to noise and overfitting, and the absence of an independent validation cohort limits the generalizability of the findings. Future studies with adequately powered sample sizes are essential to confirm the transcriptomic signatures identified herein. All mechanistic interpretations presented are hypothesis-generating and warrant experimental validation. Further, the imbalance in DWS+ and DWS- numbers makes it difficult to attribute the differences solely to infection status. Similarly, without the minimal number of replicates, the study could not explore statistical significance. Another key limitation of the present study is that all findings are derived from bulk transcriptomic profiling, and all the biological pathway observations are based on transcript abundance and do not demonstrate any pathway activation, protein expression, or functional activity, and hence should be interpreted as putative associations. Further, any inferred pathway enrichment analyses and GO annotations presented in the study are inference-based predictions and hypothesis-generating observations derived from transcriptomics data and should be interpreted as such warranting further validation.

The comparative transcriptomic profiling in the pediatric population highlights the multifaceted nature of host immune dysregulation in dengue infection. While primary pediatric dengue was characterised by altered expression of complement genes and macrophage-associated genes, secondary dengue showed a heterogeneous transcriptomic profile, with elevated immune checkpoints and altered expression of complement regulatory genes. These data show that secondary pediatric dengue is not merely an exacerbation of primary pediatric dengue alone but reflects a quantitatively distinct immune landscape, likely shaped by prior infection as well as host responses. This distinctness should, however, be interpreted with caution: the number of genes significantly differentially expressed between primary and secondary dengue (35–106 genes, depending on threshold) was substantially smaller than the number distinguishing either group from healthy controls (410–862 and 542–1340 genes, respectively; Suppl. Table 3), and the majority of complement- and macrophage-associated transcripts did not differ significantly between primary and secondary dengue (Suppl. Table 4). The clearest and most consistent divergence between the two infection groups was observed among immune checkpoint genes. Hence, we interpret primary and secondary pediatric dengue as sharing a predominantly overlapping transcriptional core, upon which a smaller, checkpoint-centred module of differences is superimposed, rather than as two wholly distinct immunological states. Importantly, the detection *C1Q* family, and checkpoint regulators *PDCD1, LAG3*, and *TIGIT* offers a translational potential for dengue diagnostics and therapeutics. In conclusion, our study underscores that severe dengue is a delicate interplay of differential antiviral defence, immune suppression, and dysregulated host metabolism, providing a foundation for precision medicine in this predominantly vulnerable population. While previous transcriptomic studies have examined dengue immune responses in adult and mixed-age cohorts, the study specifically focuses on the pediatric population, which bears a disproportionate burden of severe dengue. Although our sample size limits the scope of our conclusions, this pilot study contributes to the growing body of dengue transcriptomic literature by simultaneously examining complement pathway components, macrophage-associated markers, and immune checkpoint molecules across primary and secondary pediatric dengue infections.

In conclusion, pediatric dengue exhibits distinct systems-level immune dysregulation characterized by complement imbalance and immune checkpoint–associated exhaustion. Bulk transcriptomic profiling of PBMCs reveals that secondary dengue infection is not merely an amplified response but one that diverges from primary dengue principally within a checkpoint- and cell-cycle-associated transcriptional module superimposed on a largely shared antiviral program. These findings identify candidate pathways for targeted diagnostics and therapeutic intervention in severe dengue disease.

## Methods

### Ethical approval and assent for participation

A cross-sectional case-control study was carried out following the Guidelines of the International Conference on Harmonization and the Declaration of Helsinki (1962). The study protocols were reviewed and approved by the Institutional Ethical Committee of the Government Theni Medical College and Hospital, Theni, for conduct of the research (Ref. No. 1515/MEIII/21-4). All the human subjects were pediatric volunteers, and written assents were duly obtained from the legal guardians of all the participants. The participants were informed of the research procedures in lucidly understandable terms.

### Participants and clinical specimens

Pediatric patients diagnosed with clinical dengue infection were recruited and further stratified by IgM/IgG ratio into primary (*n* = 3) and secondary (*n* = 3) infections according to the 2009 WHO Classification. The IgM and IgG were measured using PanBio Dengue IgM (Cat. No.: 01PE20, Abbott, Chicago, IL, USA) and PanBio Dengue IgG (Cat. No.: 01PE10, Abbott, Chicago, IL, USA), respectively, and positive cut-offs were determined as per the manufacturer’s instructions. Secondary primary dengue was defined as those with IgM and IgG positivity, with an IgM/IgG ratio < 1.2, while other IgM-positive samples were considered as primary^50,51^. We also recruited age-matched pediatric healthy controls (*n* = 3). The samples were categorized into DWS+ and DWS- according to the WHO 2009 classification, at the time of admission. All enrolled participants were confirmed negative for other confounding infections, including chikungunya, leptospirosis, bacteremia, and enteric fever. Chikungunya virus-specific IgM was tested using the CHIKV MAC ELISA kit developed by the National Institute of Virology (NIV, Pune, India). Anti-leptospira IgM were tested using Panbio Leptospira IgM ELISA kit (Cat. No.: 02PE10, Abbot, Gurgaon, Haryana, India) following the manufacturer’s instructions. Blood culture was carried out using the conventional culture method for the identification of bacterial pathogens. The presence of *Salmonella typhi* or *S. paratyphi* infection was ascertained using the standard Widal agglutination test.

### Peripheral blood mononuclear cells

Two millilitres of peripheral blood samples were collected within 2-4 days of admission in hospital and a week after fever onset at the fever clinic in a PAXgene blood RNA tube (Cat. No.: 762165, Qiagen, Bangalore, India). PBMCs were isolated using SepMate^TM^ density gradient centrifugation (StemCell Technologies, Vancouver, Canada) and stained for purity using the procedure described elsewhere^52^. Briefly, an appropriate volume of Lymphoprep^TM^ was added to the SepMate^TM^ tube followed by dilution of the blood with an equal volume of PBS and 2% FBS. The diluted samples were centrifuged at 1200 g for 20 min at room temperature. The buffy coat containing the mononuclear cells was isolated and washed with PBS and 2% FBS repeatedly by a series of centrifugation steps. Cell viability was determined by 0.4% trypan blue staining on a hemocytometer. The purified PBMCs were cryopreserved in FBS + 10% DMSO and stored in liquid nitrogen in 10 million cell aliquots at −135 °C. Later, the cells were stored in Trizol (Cat. No.: 2106481001730, GeNei, Bangalore, India) before RNA extraction.

### RNA extraction, library preparation, and sequencing

RNA was extracted from PBMCs using a commercial RNeasy Mini Kit (Cat. No.: 74104, Qiagen GmbH, Germany), and quantification was performed using the Qubit RNA Assay HS (Cat. No.: Q32852, Invitrogen, MA, USA). RNA HS Screen tapes (Cat. No.: 5067-5579, Agilent, CA, USA) were used to assess the quality of RNA. Samples with a minimum RIN score of five and a concentration > 100 ng were used before processing for RNA sequencing to avoid the exclusion of clinically valuable samples, and a minimum RIN score of five was used as threshold, as this is acceptable for mRNA sequencing with ribosomal RNA depletion protocols, which are less sensitive to partial RNA degradation compared to poly(A) selection methods^53^. (While all the samples exhibited high RNA integrity (6.7–9.9), only one sample showed a RIN value near the threshold). Ribosomal RNA of cytoplasmic and mitochondrial origins was excluded using target-specific oligo beads. cDNA was synthesised from purified RNA, and the library was prepared using AMPure bead purification (Cat. No.: A63881, Beckman Coulter Life Sciences, IN, USA). RNA sequencing was done using the Illumina NovaSeq X plus platform and generated 60 M, 2 × 150 base pair reads per sample.

### RNA pre-processing and transcriptome analysis

Raw sequencing reads (FASTQ file) was processed to remove adaptors and contaminants, and was aligned, mapped. Data check was done by FastQC (ver.0.11.9), and adapter was trimmed using fastq-mcf (ver.1.05) and cutadapt (ver.4.7), while contamination removal was performed by Bowtie (ver.2.5.3). The paired ends were aligned to the human reference genome downloaded from the Gencode database (GRCh38/hg38) with alignment done by STAR (ver.2.7.11b). Distribution of mapped reads over genomic features like CDS exon, 5’UTR, and intron was performed using RSeQC and RNA-SeQC tools. For the read counts, we have used FeatureCounts (ver.2.0.6)^54^. DESeq2 (R Bioconductor package) (ver.1.38.0)^55^ was employed to identify the DEGs, and for the principal component analysis (PCA), while cufflinks (ver.2.2.1) was used for gene expression estimation. In addition, pathway and gene ontology (GO) enrichment of the differentially expressed genes were examined using ClusterProfiler (ver.3.14.3)/R DAVID web (ver.4.3.1). Pathway and functional enrichments were performed using the Qiagen Ingenuity Pathway Analysis (IPA) (version as applicable) (Qiagen Inc., Redwood City, CA, USA)

### RNA sequencing

mRNA sequencing was completed for all the nine samples. An average of 80 million paired-end reads per sample were generated (2 × 150 bp read length), with an average G + C content of 46%. All samples yielded high-quality RNA sequencing data with relatively good alignment rates and comprised gene expression profiles. Contextual analysis was completed for 11,887 genes across four pair-wise comparisons: Healthy controls versus primary dengue and secondary dengue (HC vs P + S dengue), healthy controls versus primary dengue (HC vs P dengue), healthy control versus secondary dengue (HC vs S dengue), and primary dengue versus secondary dengue (P vs. S dengue). Hierarchical clustering analysis revealed distinctions in sample groups, providing validity to the experimental design, and clear transcriptional signatures in relation to progression with state (Suppl. Fig. 1).

### Data normalization

Raw gene counts were normalised and transformed into log₂ counts per million (log₂-CPM) values to adjust for variations in library size and RNA composition between the samples. Quality assessment of the normalised data was performed, all samples showed similar dynamic ranges of gene expression, with log₂-CPM values spanning from −2 to 12, and comparable numbers of outlier genes (Suppl. Fig. 2). The uniform distribution characteristics across healthy controls (H1–H3), primary dengue (P1, P2, P5), and secondary dengue samples (S1–S3) confirm that any observed differences in subsequent analyses will be genuine biological variation rather than technical errors.

### Statistical analysis

All analyses for the clinico-demographic details were performed using the Mann-Whitney U test (two-tailed test), with exact *p*-values reported; significance was set at *p* ≤ 0.05. The descriptive analysis was presented as mean ± SD owing to the smaller sample size. Data were compiled in MS Excel ver.14.4.9, (Microsoft Corp., USA). Statistical analyses for the DEGs were performed by DESeq2 (ver.1.38.0), which estimates dispersion, applies shrinkage for low-count genes, and provides regularised logarithmic (rlog) values for robust differential expression assessments. Multiple testing correction was performed using the Benjamini-Hochberg method as implemented in DESeq2, with an adjusted *p*-value (FDR) < 0.05 and |log₂FC | ≥ 2 as the significance thresholds for DEG identification. All nine samples were sequenced in a single batch on the Illumina NovaSeq X Plus platform, and therefore batch correction was not required.

## Supplementary information

Supplementary Items.

## Data Availability

All the datasets used in the manuscript are publicly available. The RNA sequencing fastq files and corresponding raw counts for transcriptomics data generated in this study are available at https://www.ncbi.nlm.nih.gov/bioproject/PRJNA1453950.
