# Supplementary material for: Bulk transcriptomics of peripheral blood mononuclear cells delineates systems-level immune dysregulation in pediatric dengue infection
Source: NPJ Syst Biol Appl. 2026 Aug 14;12:123. doi: 10.1038/s41540-026-00796-y (PMC13477468; doi:10.1038/s41540-026-00796-y)
Supplement: Supplementary file 1 [file 41540_2026_796_MOESM1_ESM.pdf]

Bulk transcriptomics of peripheral blood mononuclear cells delineates systems-level immune dysregulation in pediatric dengue infection

Abdul R Anshad[1], Muthuvel Atchaya[1], Amudhan Murugesan[2], Suvaiyarasan Suvaithenamudhan[3], Shanmugam Saravanan[4], Sivadoss Raju[5], Cecilia Svanberg[6], Marie Larsson[6*], Esaki M Shankar[1*]

[1]Infection and Inflammation, Department of Biotechnology, Central University of Tamil Nadu, Neelakudi, Thiruvarur 610 005, India, [2]Department of Microbiology, The Government Theni Medical College and Hospital, Theni, India, [3]School of Biosciences and Bioengineering, Dr. D. Y. Patil International University, Akurdi, Pune 411044, Maharashtra, India, [4]Genomics Infectious Diseases Research, Dr. D. Y. Patil Dental College and Hospital, Pimpri, Pune 411018, Maharashtra, India, [5]State Public Health Laboratory, Directorate of Public Health and Preventive Medicine, DMS Campus, Chennai, India, [6]Molecular Medicine and Virology, Department of Biomedical and Clinical Sciences, Linköping University, Linköping 58 185, Sweden.

Supplementary Figures

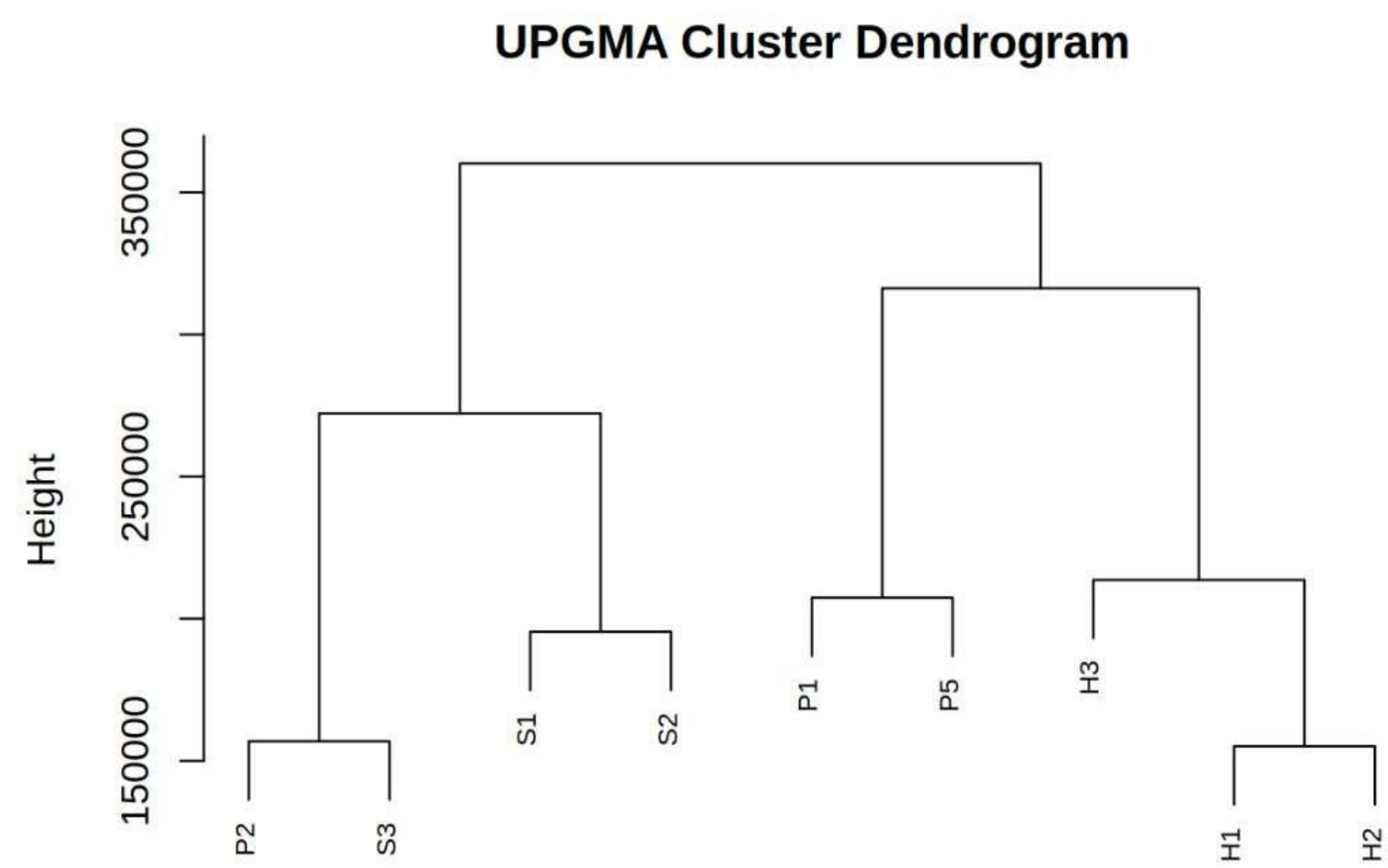

Supplementary Fig. 1: Hierarchical clustering of study samples using UPGMA clustering dendrogram from expression of protein coding genes. The unweighted pair group method with arithmetic mean (UPGMA) dendrogram depict the hierarchical relation between healthy, primary, and secondary samples based on transcriptomic expression profile.

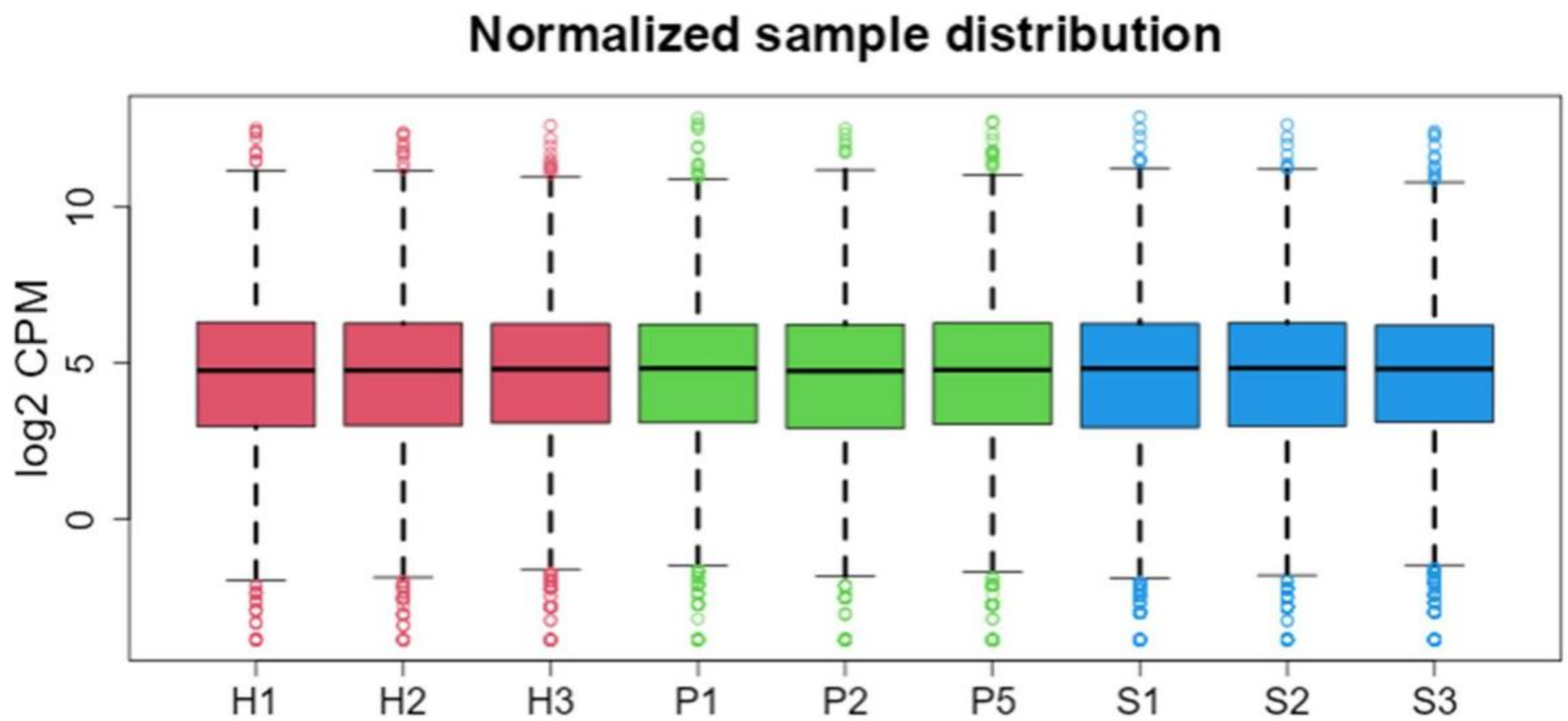

Supplementary Fig. 2: Normalized sample distribution show good quality of samples. The distribution of $\log_2$ counts per million (CPM) values for each samples after normalization of gene expression was assessed. Samples are grouped by condition: healthy controls (H1–H3, red), primary dengue infection (P1, P2, P5, green), and secondary dengue infection (S1–S3, blue). The black horizontal lines indicate medians, boxes represent interquartile ranges (IQR), and whiskers extend to 1.5 × IQR, and individual points show outlier values.

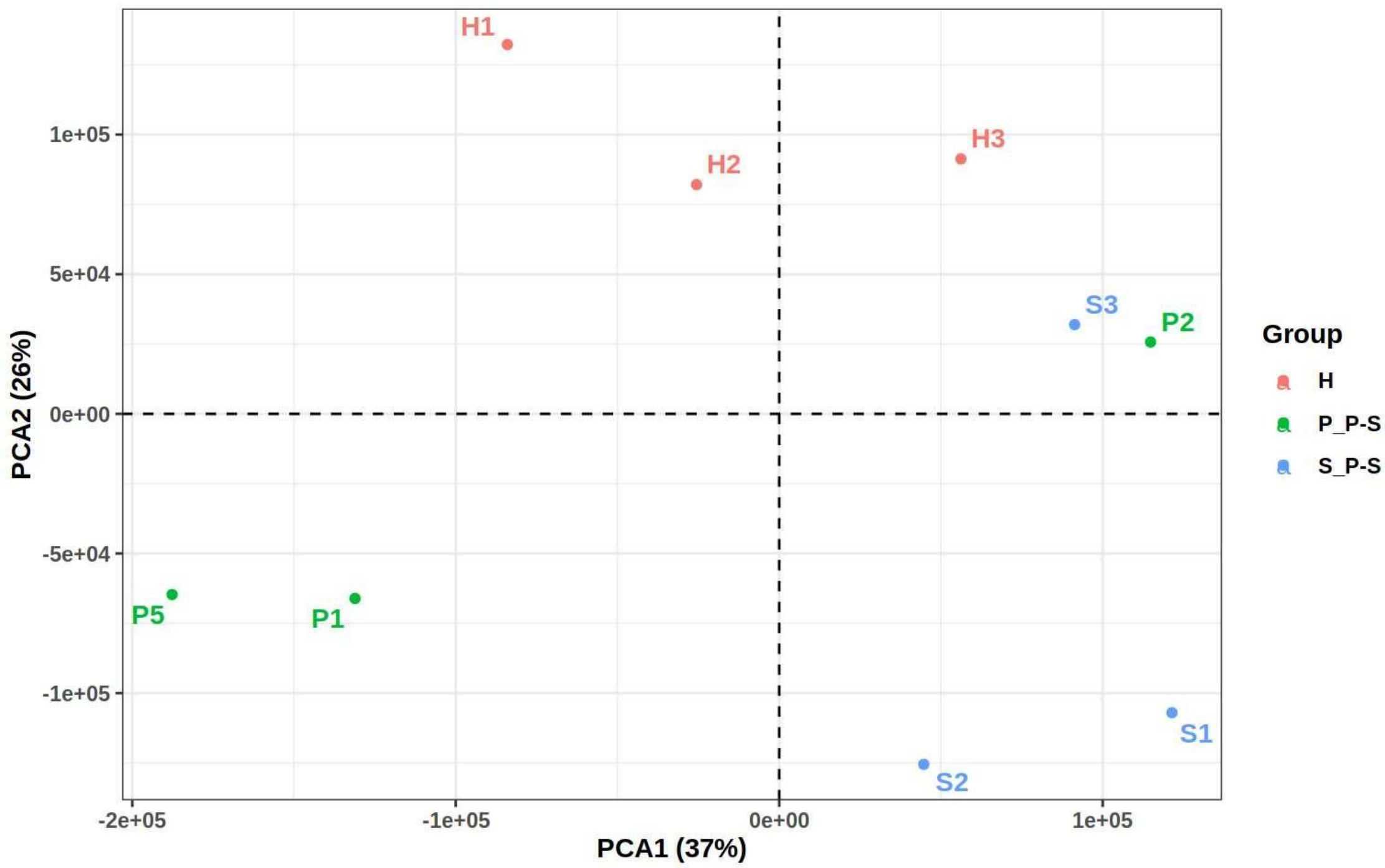

Supplementary Fig. 3: Principal component analysis (PCA) of transcriptomes across DENV infected individuals and healthy controls. The gene expression profiles from healthy controls (H1–H3, red), primary dengue infection (P1, P2, P5, green), and secondary dengue infection (S1–S3, blue). Each point represents an individual sample.

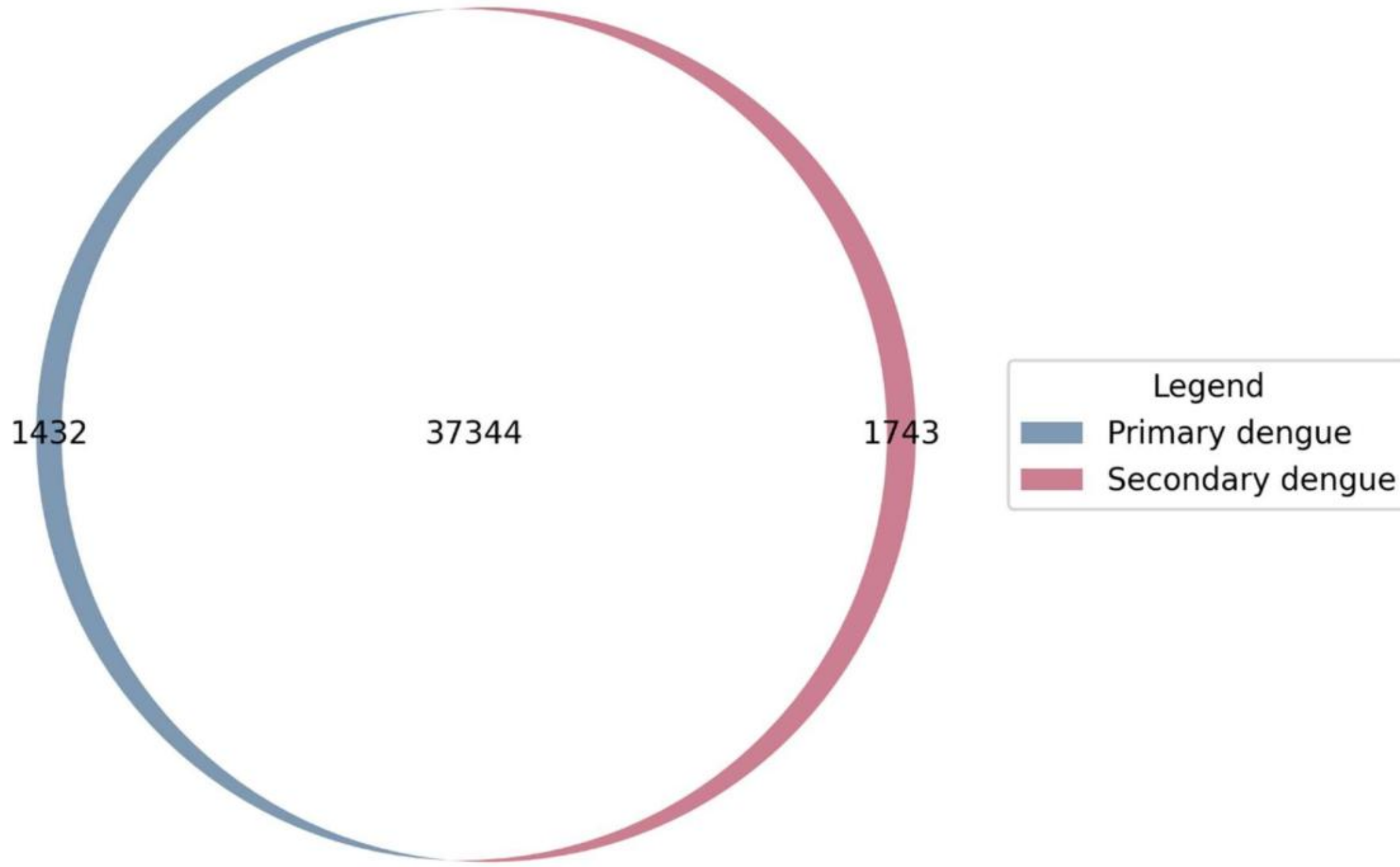

Supplementary Fig. 4: Comparative transcriptomic profiling of primary and secondary pediatric dengue infections. Venn diagram depicting the overlapping of DEGs between primary and secondary pediatric dengue.

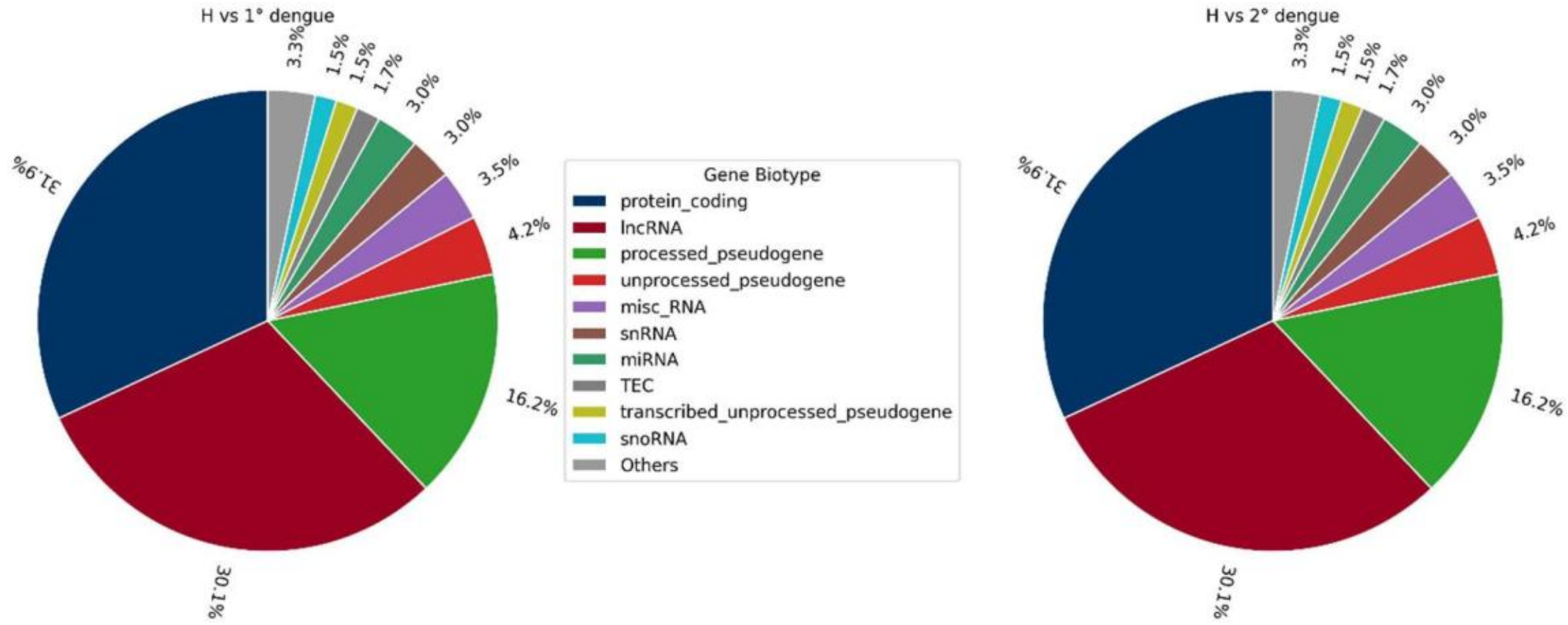

Supplementary Fig. 5: The Pie chart represents the distribution of genes based on the gene biotypes across the primary and secondary pediatric dengue. The gene biotypes were classified based on the ensembled genotype into protein-coding, lncRNA, pseudogenes, snRNA, etc.

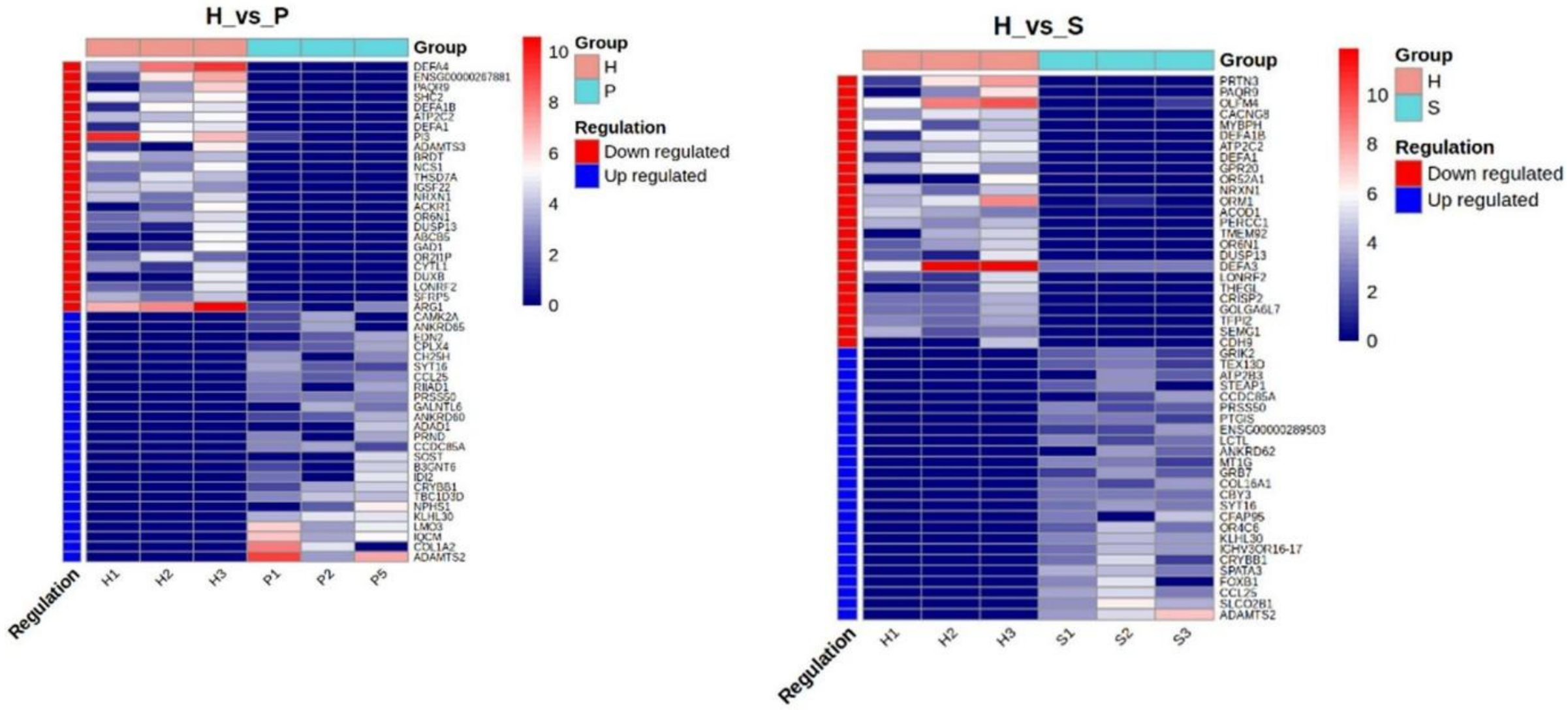

Supplementary Fig. 6: Differentially expressed top 25 upregulated and downregulated gene transcriptomes across the study cohorts. A) The top 25 upregulated and 25 downregulated significantly differentially expressed protein-coding genes in primary (P) vs healthy controls (HC). B) The top 25 upregulated and 25 downregulated significantly differentially expressed protein-coding genes in secondary (S) vs healthy controls (HC) expression for the top 25 and bottom 25 significantly differentially expressed protein-coding genes.

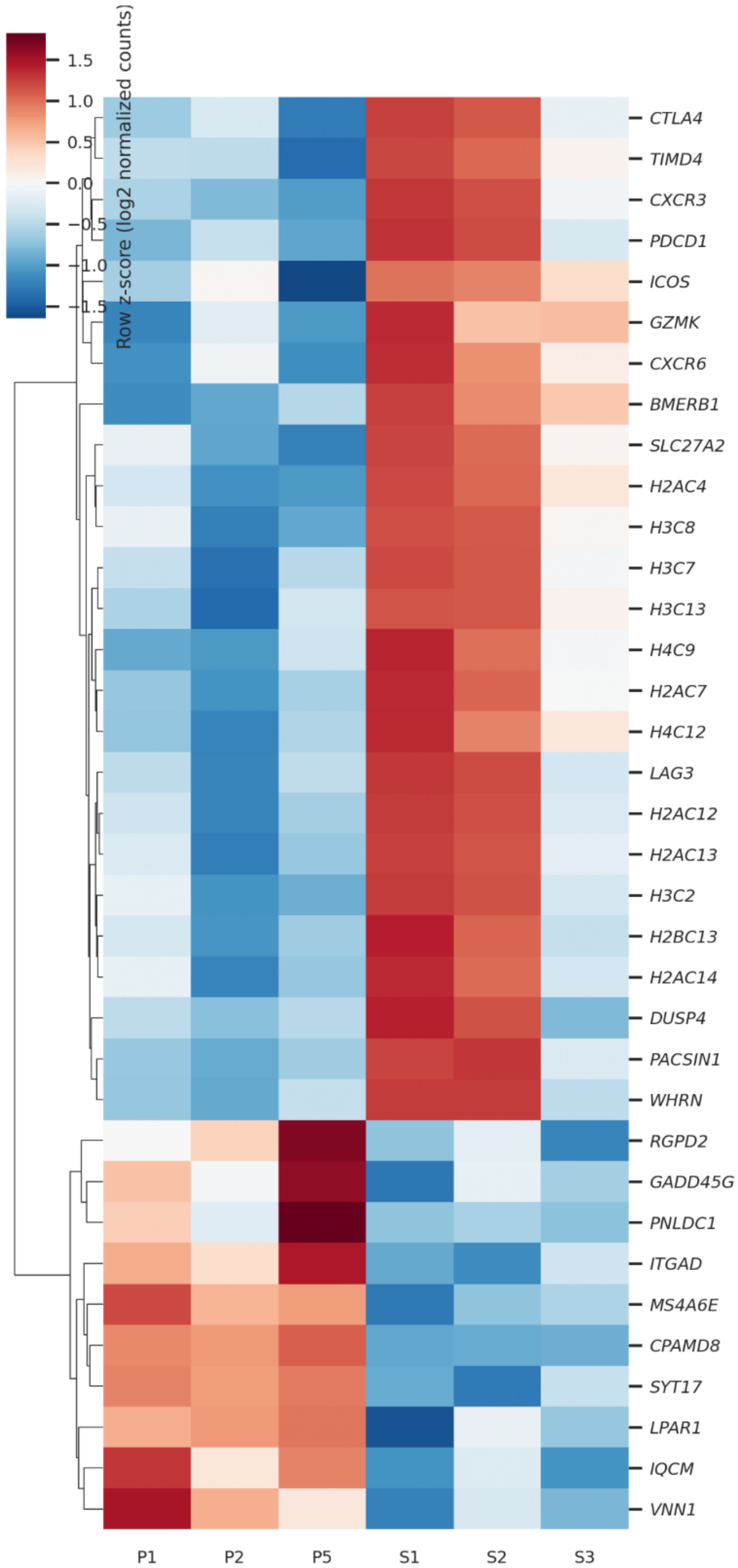

Supplementary figure 7. Heat map of 35 genes significantly differentially expressed between primary and secondary pediatric dengue (FDR<0.05, |logFC|≥2; DESeq2). Rows show row-wise Z scores of log2(normalized counts+1); columns are individual patients. Hierarchical clustering (rows) cleanly separates primary from secondary dengue (column order fixed by group). Genes includes immune-check points transcripts (CLT4, PDCD1< ICOS, LAG3) and cell-cycle/histone genes (H2AC4, H3C7, H4C9, etc.) consistent with check points and proliferation-associated divergence in the text

Supplementary Tables

Supplementary Table 1: Baseline demographic, clinical and biochemical characteristics of pediatric dengue patients, with comparison between primary and secondary pediatric dengue infections. Baseline demographic and clinical characteristics of the dengue patients (n=6). Variables accessed included minimum, maximum and mean values for the age, and number of DWS+ and DWS- patients, as well as gender. Values expressed as mean±s.d. Statistical analysis was done using the non-paired t test, p<0.05 was considered significant. Abbreviations: ALP, alkaline phosphatase; ALT, alanine aminotransferase; AST, aspartate aminotransferase; Hb, hemoglobin; PCV, packed cell volume; WBC, white blood cell. *Indicates p<0.05. †Reference intervals were extracted from the clinical sample collection unit of the hospital

**Variables**	n=6
Age (in years)	
Min	3
Max	11
Average age primary dengue	6
Average age Secondary dengue	9
**Dengue classification**	
DWS+	5
DWS-	1
**Gender**	
Male	5
Female	1

**Laboratory parameters**	**Reference interval†**	**Primary (n=3)**	**Secondary (n=3)**	**P value**
**Respiratory rate (/min)**	20-30	26±0	24±3.46	0.422
**Hb (g/dL)**	11-14	11.07±1.46	12.87±0.57	0.117
**PCV**	33-40%	35.37±2.6	36.67±1.24	0.478
**Platelet (/uL)**	150-100x10^3	181±113.53	63.33±55.14	0.181
**WBC count (/uL)**	5000-10000	10600±7767.24	5500±1452.58	0.326
**AST (U/L)**	<40	71±33.81	172.67±70.19	0.086
**ALT (U/L)**	<40	62±48	85.33±49	0.587
**ALP (U/L)**	50-150	138.33±50.82	143.33±81.21	0.932
**Albumin (g/dL)**	3.5-5.0	4.13±1.02	3.13±0.29	0.7
**Urea (mg/dL)**	7-20	25.33±5.51	20.67±3.06	0.268
**Creatinine (mg/dL)**	0.3-0.8	0.83±0.06	0.83±0.06	1
**Sodium (mmol/L)**	135-145	132.33±2.52	132.67±2.08	0.868

Supplementary Table 2: Significant differential expression analysis without shrinks in fold-change for protein-coding genes. Summary of differential gene expression analysis. A cut-off of $p<0.05$ and fold change gene expression ≤ -2 or ≥+2 was employed. P refers to primary dengue; S refers to secondary dengue and HCs refer to healthy controls.

Control Vs Case group	Total genes	Upregulated	Downregulated
**P+S vs HCs**	1618	916	702
**P vs HCs**	1539	746	793
**S vs HCs**	1937	1012	925
**S vs P**	1015	519	496

Supplementary Table 3: Summary of Differential expression and overlap across clinical comparisons. Differentially expressed genes were identified using DESeq2. Upregulated and downregulated genes were defined accordingly. False discovery rate (FDR) was controlled using the Benjamini-Hochberg method. Overlap analysis was performed using the stringent DEGs from healthy vs primary and healthy vs secondary comparisons.

**Comparison**	**Threshold**	**Total significant genes**	**Upregulated**	**Downregulated**	**Median |log2FC| (genes with padj<0.05)**
Healthy vs Primary	FDR<0.05, |log2FC|≥2	410	243	167	1.51
Healthy vs Primary	FDR<0.05, |log2FC|≥1	862	520	342	
Healthy vs Primary	nominal p<0.05, |log2FC|≥1	1618	916	702	
Healthy vs Secondary	FDR<0.05, |log2FC|≥2	542	334	208	1.33
Healthy vs Secondary	FDR<0.05, |log2FC|≥1	1340	788	552	
Healthy vs Secondary	nominal p<0.05, |log2FC|≥1	1937	1012	925	
Primary vs Secondary	FDR<0.05, |log2FC|≥2	35	25	10	1.82
Primary vs Secondary	FDR<0.05, |log2FC|≥1	106	76	30	
Primary vs Secondary	nominal p<0.05, |log2FC|≥1	1015	519	496	

Overlap between Healthy-vs-Primary and Healthy-vs-Secondary DEG sets (FDR<0.05, |log2FC|≥2):

Genes shared (overlap)	346
Union	606
Jaccard overlap (%)	57.1%
% of H-vs-P DEGs also significant in H-vs-S	84.4%
% of H-vs-S DEGs also significant in H-vs-P	63.8%

Supplementary Table 4: module-level differential expression summary across clinical comparison. Module-level analysis using predefined gene sets representing the complement, macrophage acute phase, and immune checkpoints. These data show that the complement and macrophage/acute-phase modules carry no FDR-significant primary-vs-secondary differences, while the immune-checkpoint module is significantly and consistently divergent

**Module**	**Comparison**	**Genes tested**	**N significant**	**% upregulated**
Complement	H vs P	18	4	61.1%
Complement	H vs S	18	3	50.0%
Complement	P vs S	18	0	16.7%
Macrophage/Acute-phase	H vs P	9	0	55.6%
Macrophage/Acute-phase	H vs S	9	0	33.3%
Macrophage/Acute-phase	P vs S	9	0	22.2%
Immune checkpoint	H vs P	8	1	75.0%
Immune checkpoint	H vs S	8	3	100.0%
Immune checkpoint	P vs S	8	3	87.5%
